# Supplementary material for: Robust enzyme discovery and engineering with deep learning using CataPro
Source: Nat Commun. 2025 Mar 20;16:2736. doi: 10.1038/s41467-025-58038-4 (PMC11923063; doi:10.1038/s41467-025-58038-4)
Supplement: Supplementary file 1 — Supplementary Information [file 41467_2025_58038_MOESM1_ESM.pdf]

Supplementary Information

Zechen Wang[1], Dongqi Xie[2], Dong Wu[2], Xiaozhou Luo[3,4,5], Sheng Wang[2],

Yangyang Li[1], Yanmei Yang[6], Weifeng Li[1], Liangzhen Zheng[2,7]

[1] School of Physics, Shandong University, Jinan, 250100, Shandong, China.

[2] Shanghai Zelixir Biotech Co. Ltd, Shanghai, 201210, Shanghai, China.

[3] Shenzhen Key Laboratory for the Intelligent Microbial Manufacturing of Medicines, Shenzhen Institute of Advanced Technology, Chinese Academy of Sciences, Shenzhen, 518055, Guangdong, China.

[4] Key Laboratory of Quantitative Synthetic Biology, Shenzhen Institute of Advanced Technology, Chinese Academy of Sciences, Shenzhen, 518055, Guangdong, China.

[5] Center for Synthetic Biochemistry, Shenzhen Institute of Advanced Technology, Chinese Academy of Sciences, Shenzhen, 518055, Guangdong, China.

[6] College of Chemistry, Chemical Engineering and Materials Science, Key Laboratory of Molecular and Nano Probes, Ministry of Education, Shandong Normal University, Jinan, 250014, Shandong, China.

[7] Shenzhen Zelixir Biotech Co. Ltd, Hengtaiyu Park, Shenzhen, 518107, Guangdong, China.

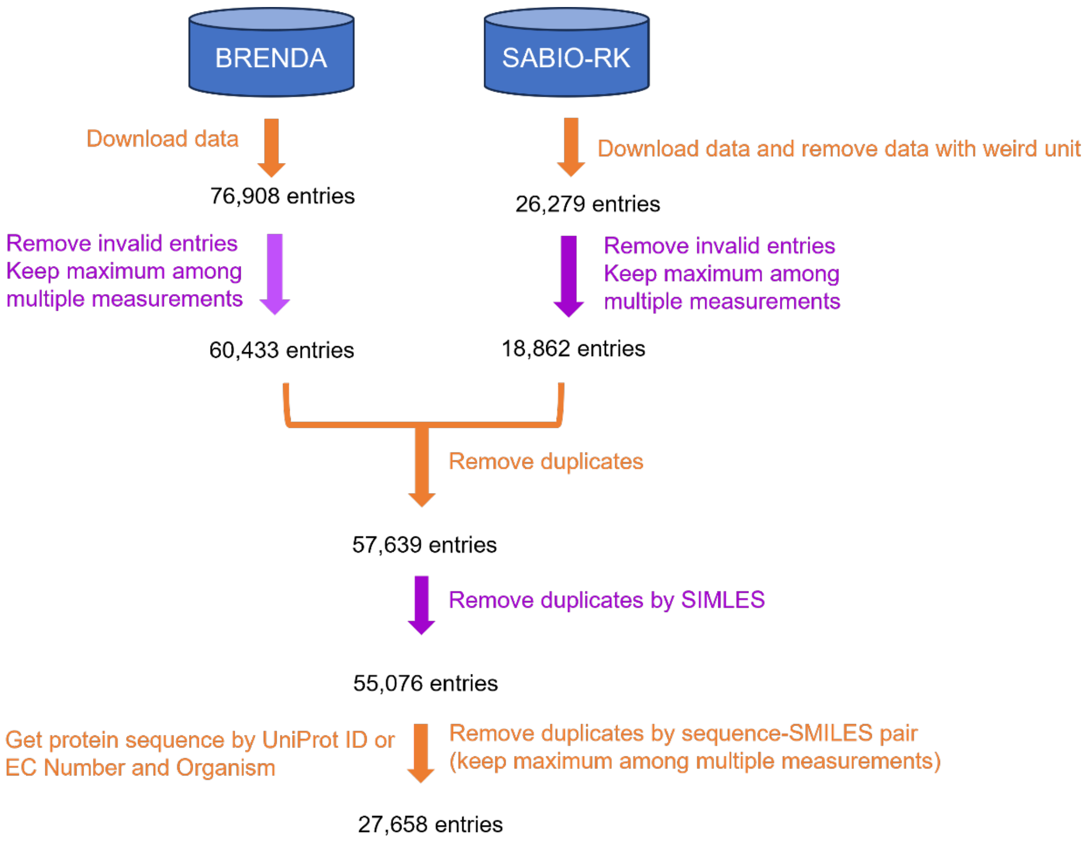

Supplementary Figure 1. The collection and cleaning process for k_{cat} entries.

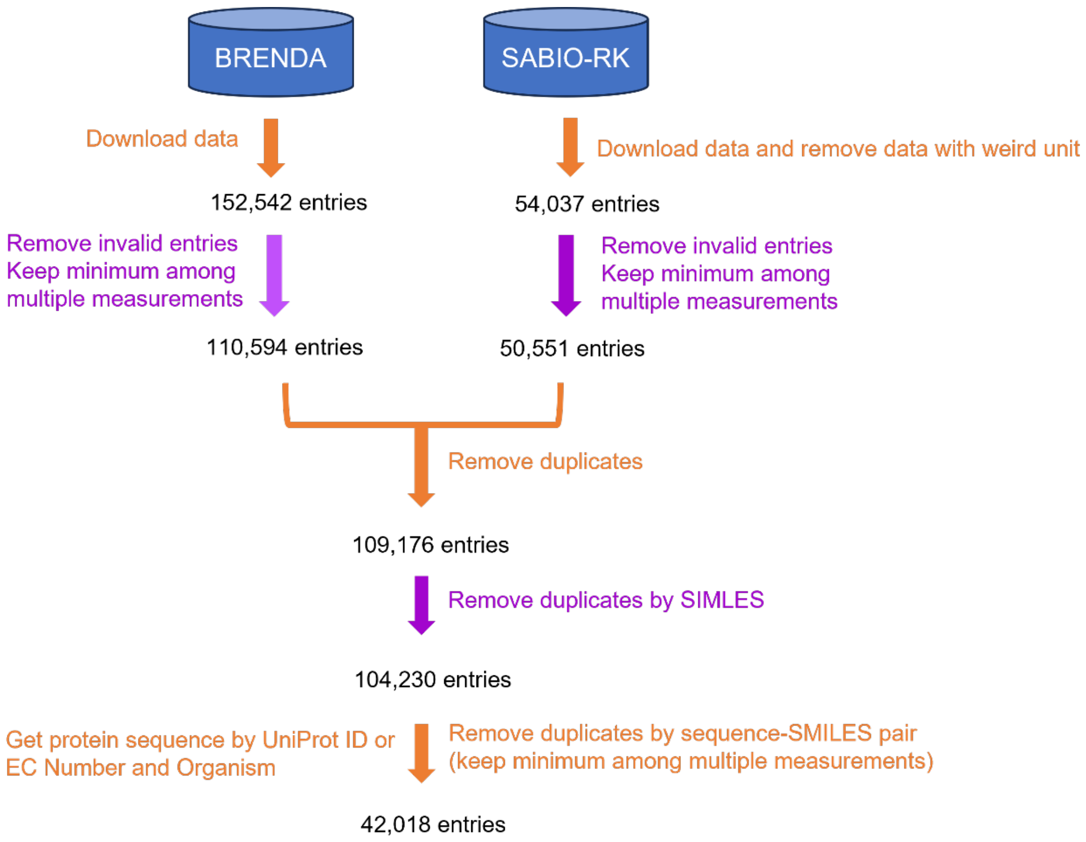

Supplementary Figure 2. The collection and cleaning process for K_m entries.

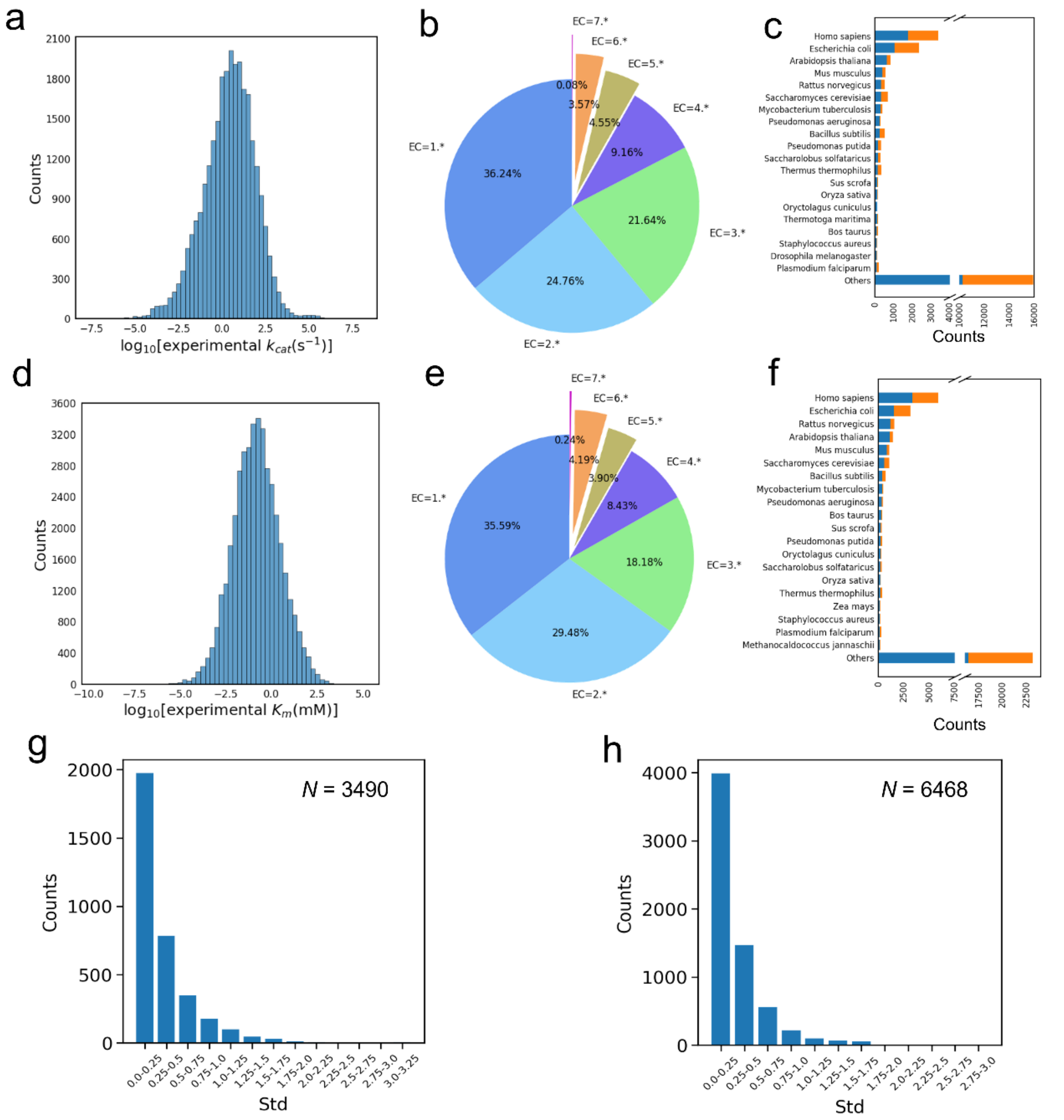

Supplementary Figure 3. Analysis of the processed k_{cat} and K_m data. a,d Distribution of k_{cat} and K_m values. **b,e** Classification of enzymes in the k_{cat} and K_m datasets based on the first digit of the EC number. **c,f** Species distribution of data in the k_{cat} and K_m datasets. **g,h** Distribution of samples across different label noise intervals in the k_{cat} and K_m databases. "Std" represents the standard deviation. Source data are provided as a Source Data file.

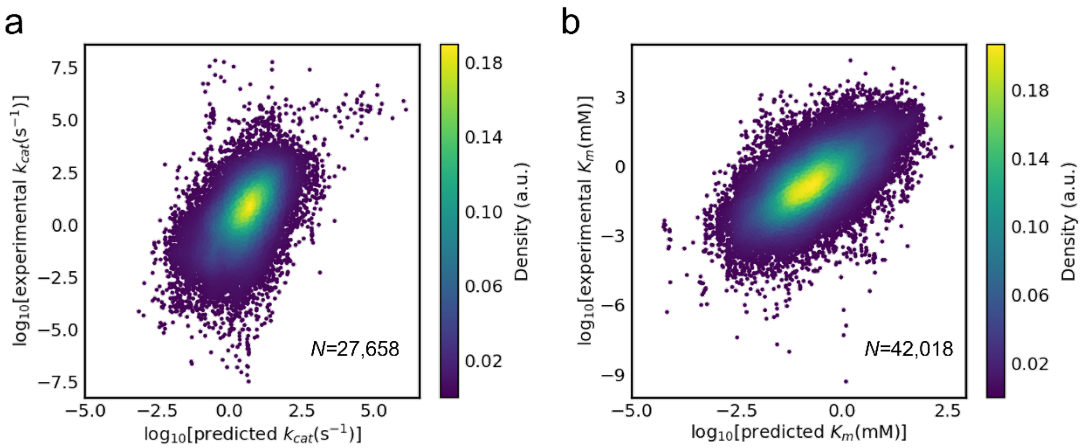

Supplementary Figure 4. Scatter plots of predicted values from CataPro and experimental values on the (**a**) k_{cat} and (**b**) K_m datasets. Source data are provided as a Source Data file.

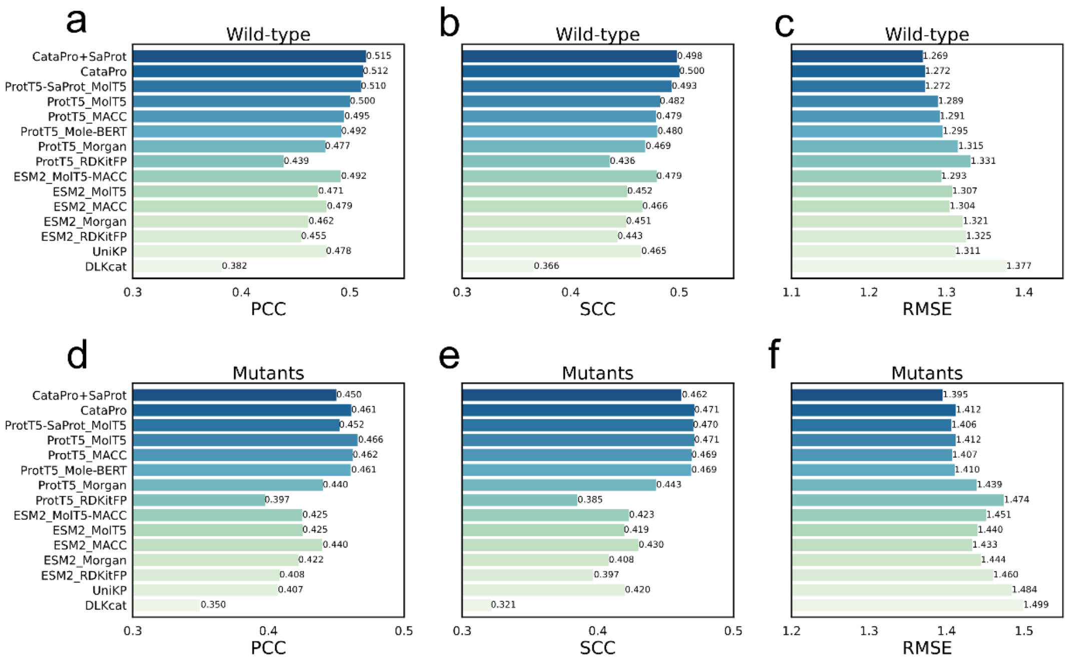

Supplementary Figure 5. Comparison of models on the k_{cat} wild-type and mutant subsets. a-c Performance of models on the wild-type samples in the k_{cat} dataset in terms of PCC, SCC, and RMSE. **d-f** Performance of the models on the mutant enzyme samples in the k_{cat} dataset in terms of PCC, SCC, and RMSE. Source data are provided as a Source Data file.

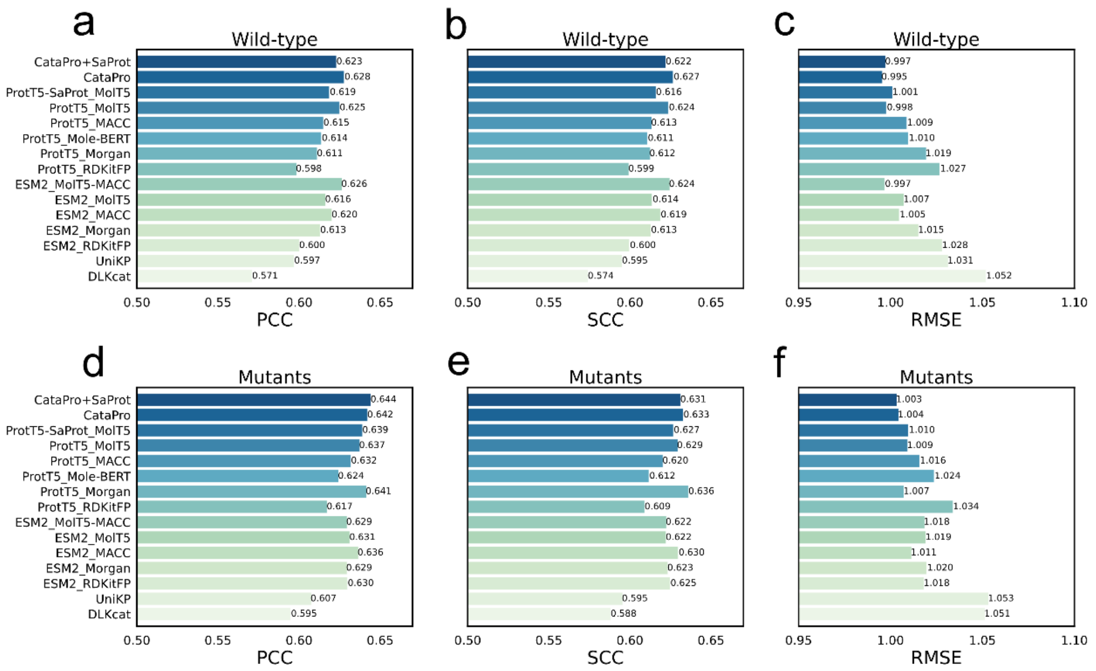

Supplementary Figure 6. Comparison of models on the K_m wild-type and mutant subsets. a-c Performance of models on the wild-type samples in the K_m dataset in terms of PCC, SCC, and RMSE. **d-f** Performance of the models on the mutant enzyme samples in the K_m dataset in terms of PCC, SCC, and RMSE. Source data are provided as a Source Data file.

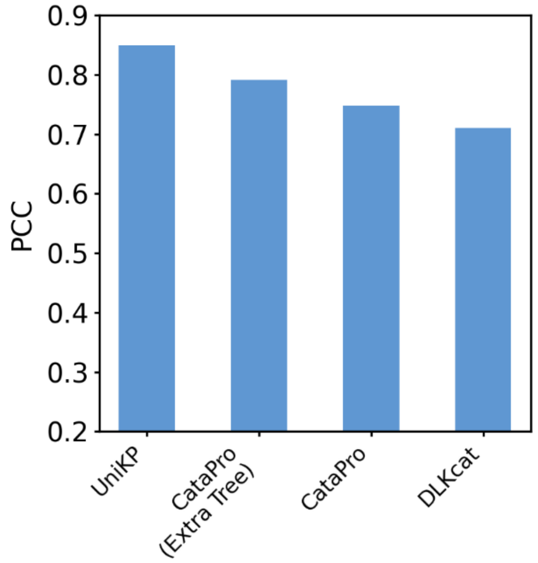

Supplementary Figure 7. Performance of CataPro on the k_{cat} dataset collected by Li et al. In accordance with the literature, 1,684 samples were randomly selected as the test set, with the remaining samples used for training and validation of CataPro. The Pearson correlation coefficients (PCC) achieved by CataPro and CataPro (extra tree) were 0.748 and 0.791, respectively, compared to 0.71 and 0.85 reported for DLKcat and UniKP. Source data are provided as a Source Data file.

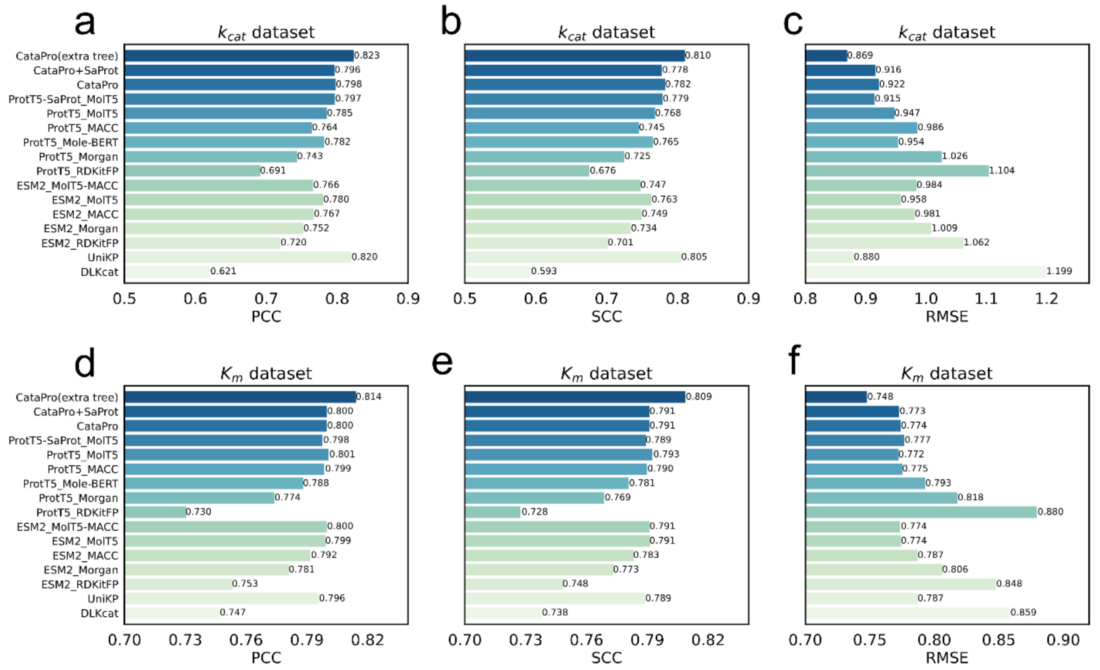

Supplementary Figure 8. Comparison of models on randomly-split 10-fold cross-validation datasets of k_{cat} and K_{m}. a-c PCC, SCC, and RMSE achieved by models on the k_{cat} dataset. **d-f** PCC, SCC, and RMSE achieved by models on the K_{m} dataset. CataPro (extra tree) represents the use of extra trees, instead of neural networks, to fit the features of CataPro and labels. Source data are provided as a Source Data file.

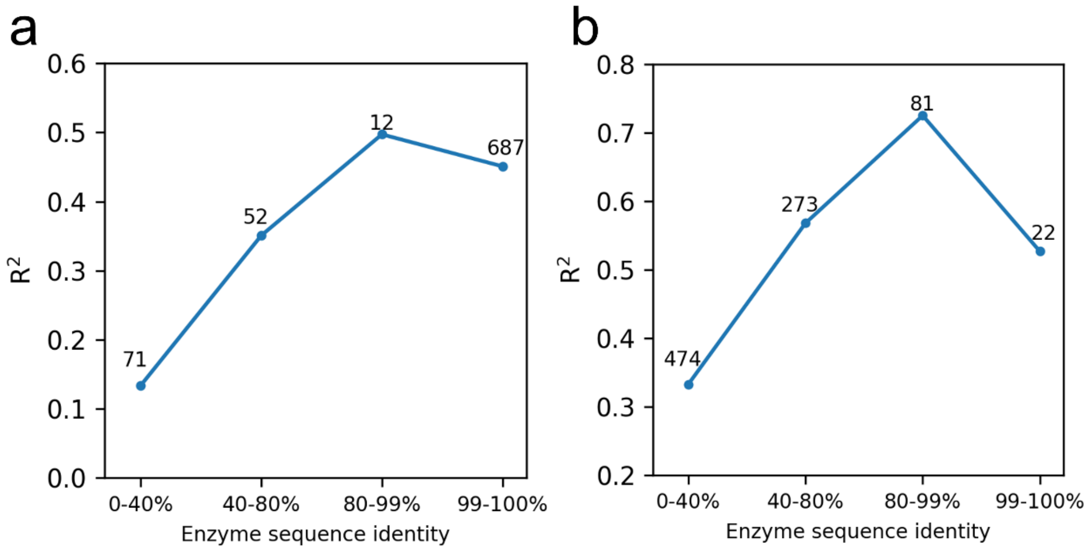

Supplementary Figure 9. Performance of CataPro and the retrained CataPto on the TurNup test set. a The performance of CataPro on the TurNuP test set across four subsets, which were divided based on the maximum sequence identity to enzyme sequences in the CataPro k_{cat} dataset. **b** The performance of the retrained CataPro on the four subsets of the TurNuP test set, which are consistent with those in the original TurNuP paper. The number of samples in each subset is shown above each point. Source data are provided as a Source Data file.

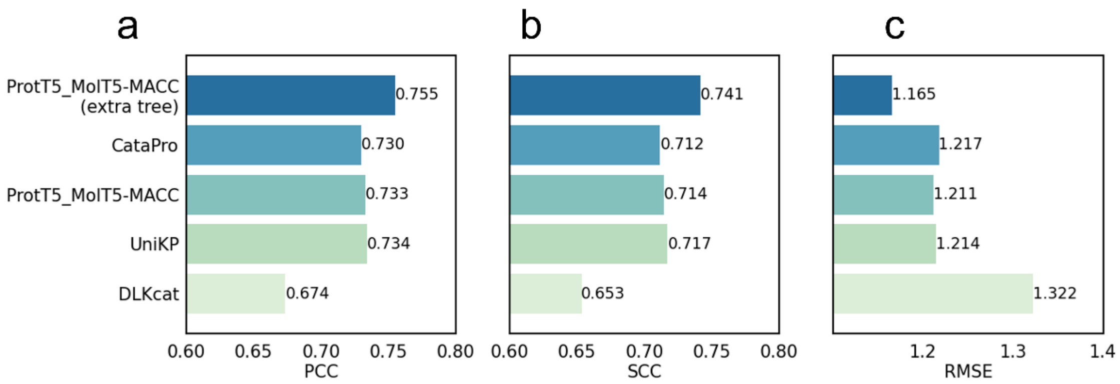

Supplementary Figure 10. Performance of models on randomly-split 10-fold cross-validation datasets of k_{cat}/K_m. a-c show the PCC, SCC, and RMSE achieved by models, respectively. ProtT5_MolT5-MACC (extra tree) represents the use of extra trees, instead of neural networks, to fit the features of "ProtT5+MolT5+MACC" and labels. Source data are provided as a Source Data file.

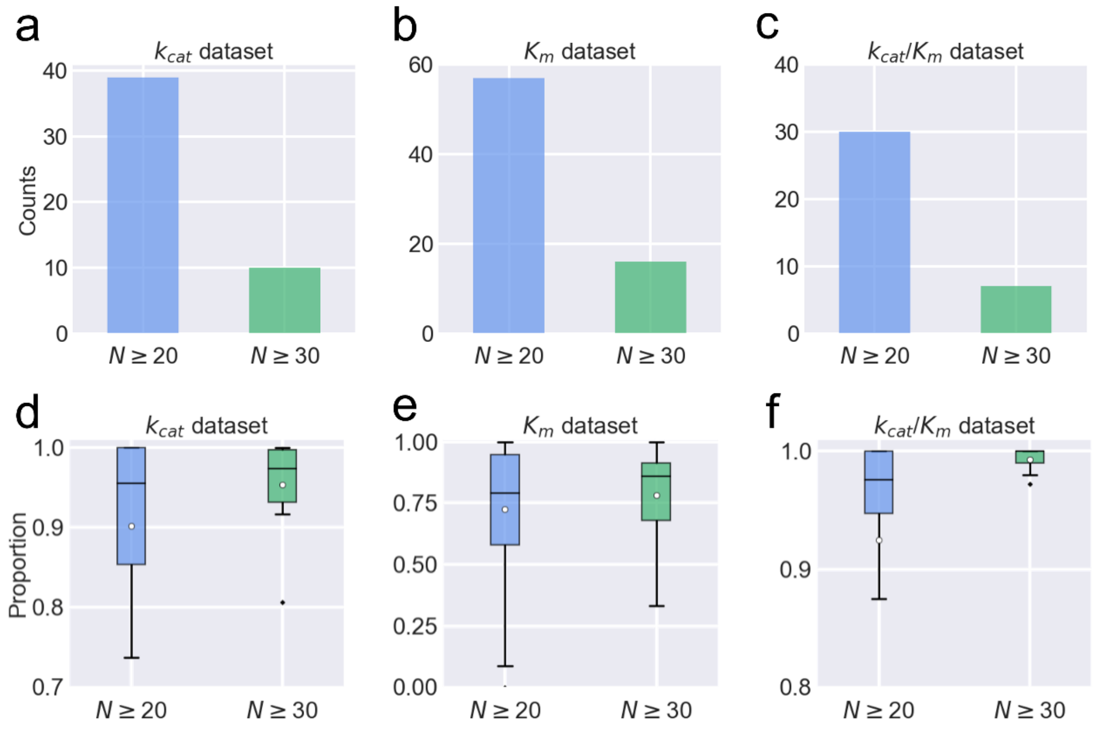

Supplementary Figure 11. Statistics on the number of reactions in the three datasets with more than 20 or 30 mutants and the proportion of disadvantageous mutants. a-c The number of reactions that meet the criteria $N \geq 20$ or $N \geq 30$ in the k_{cat}, K_m, and k_{cat}/K_m datasets. **d-f** The proportion distribution of disadvantageous mutations among all mutants in a reaction (with the same UniProtID-SMILES pair) that meets the criteria $N \geq 20$ or $N \geq 30$ in the k_{cat}, K_m, and k_{cat}/K_m datasets. In panels d-f, the lower and upper boundaries of the box represent the first quartile (Q1) and the third quartile (Q3), respectively. The whiskers extend from the quartiles to the minimum and maximum values within 1.5 times the interquartile range. The white circle represents the mean value of each statistic and the black line inside the box represents the median. Source data are provided as a Source Data file.

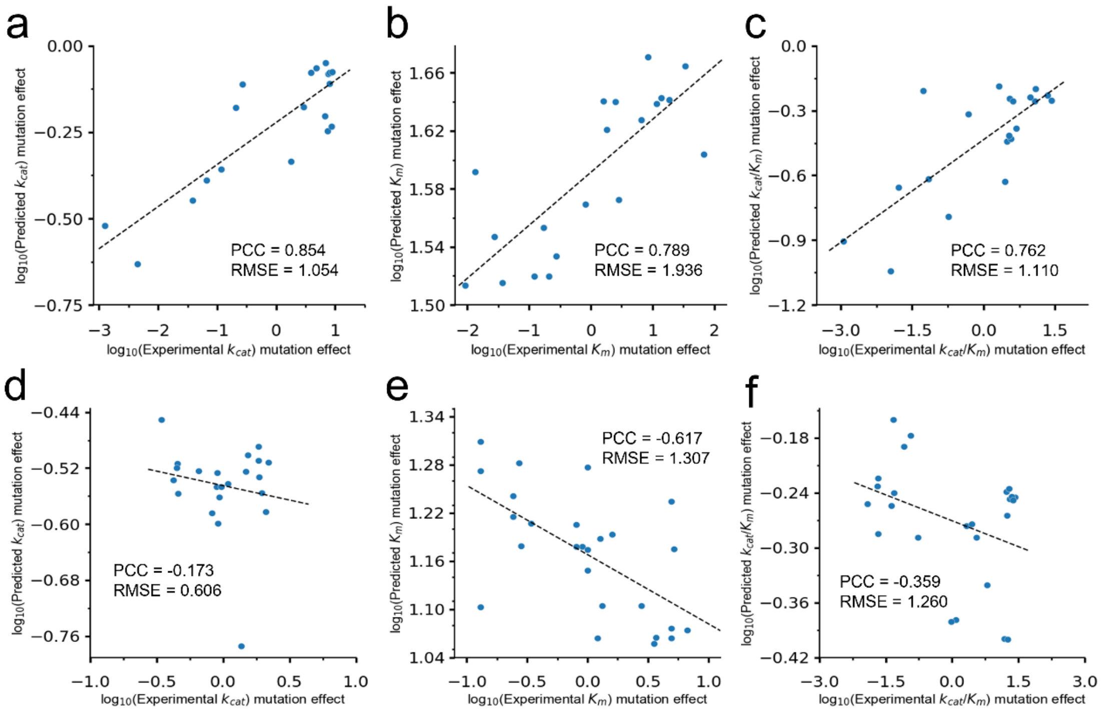

Supplementary Figure 12. Predictions of CataPro in representative enzymatic reactions. **a**, **b**, and **c** show the enzymatic reactions with the highest correlation in predicting mutation effects by CataPro on the k_{cat}, K_{m}, and $k_{\mathrm{cat}}/K_{\mathrm{m}}$ datasets, with the corresponding enzyme (UniProt ID)-substrate pairs being (Q9UKK9, ADP-D-ribose), (P50384, Anthranilate), and (Q9UKK9, ADP-D-ribose), respectively. **d**, **e**, and **f** show the enzymatic reactions with the lowest correlation in predicting mutation effects by CataPro on the k_{cat}, K_{m}, and $k_{\mathrm{cat}}/K_{\mathrm{m}}$ datasets, with the corresponding enzyme (UniProt ID)-substrate pairs being (P13956, S-Adenosyl-L-methionine), (P26276, alpha-D-Glucose 1-phosphate), and (P51570, ATP), respectively. Source data are provided as a Source Data file.

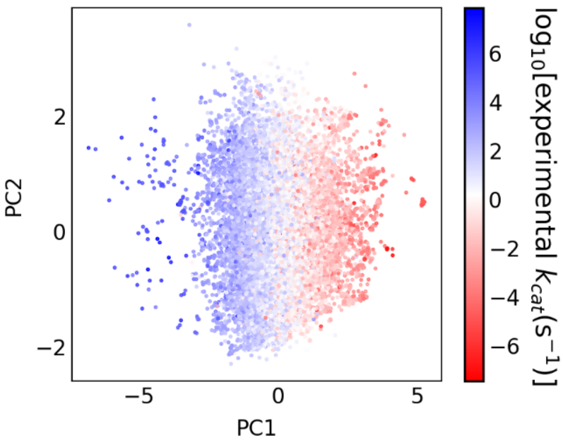

Supplementary Figure 13. Principal component analysis of the embedding extracted by the k_{cat} model. The color bar represents the experimentally measured k_{cat} values. Source data are provided as a Source Data file.

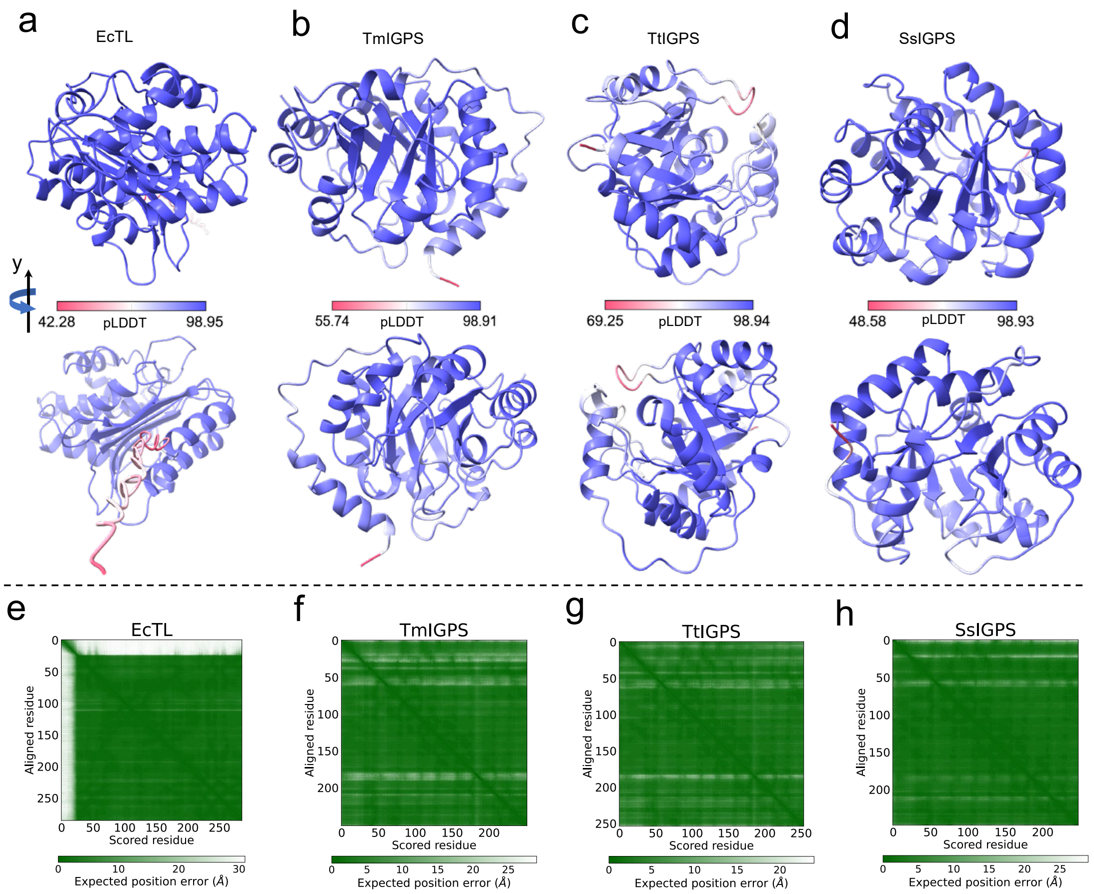

Supplementary Figure 14. Accuracy of protein structures in the DMS datasets generated by AlphaFold2. a-d respectively show the pLDDT of EcTL, TmIGPS, TtIGPS, and SsIGPS from two perspectives, with the lower view in each panel obtained by rotating the upper view 180 degrees around y-axis. **e-h** show the Predicted Aligned Error (PAE) of these four AlphaFold2 structures. The structures, pLDDT, and PAE of these four proteins are all from the AlphaFold Database.

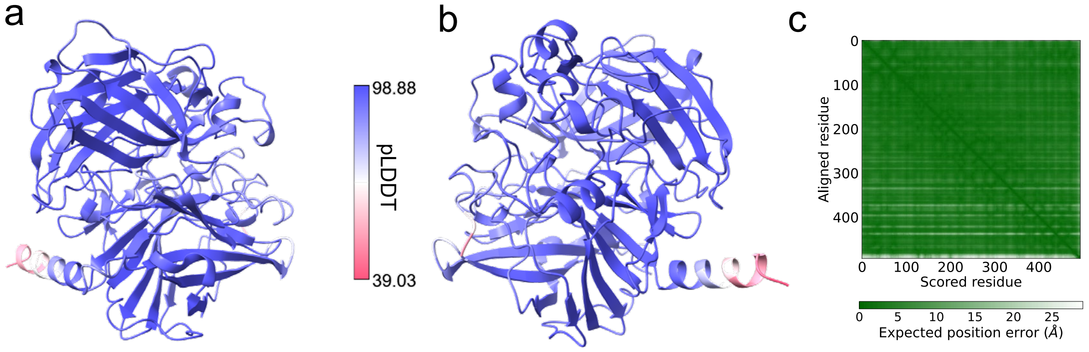

Supplementary Figure 15. Accuracy of SsCSO protein structures generated by AlphaFold2. a,b show the pLDDT from two perspectives. The view in panel **b** is obtained by rotating the view in panel **a** 180 degrees around the y-axis. **c** shows the Predicted Aligned Error (PAE) of the SsCSO AlphaFold2 structure.

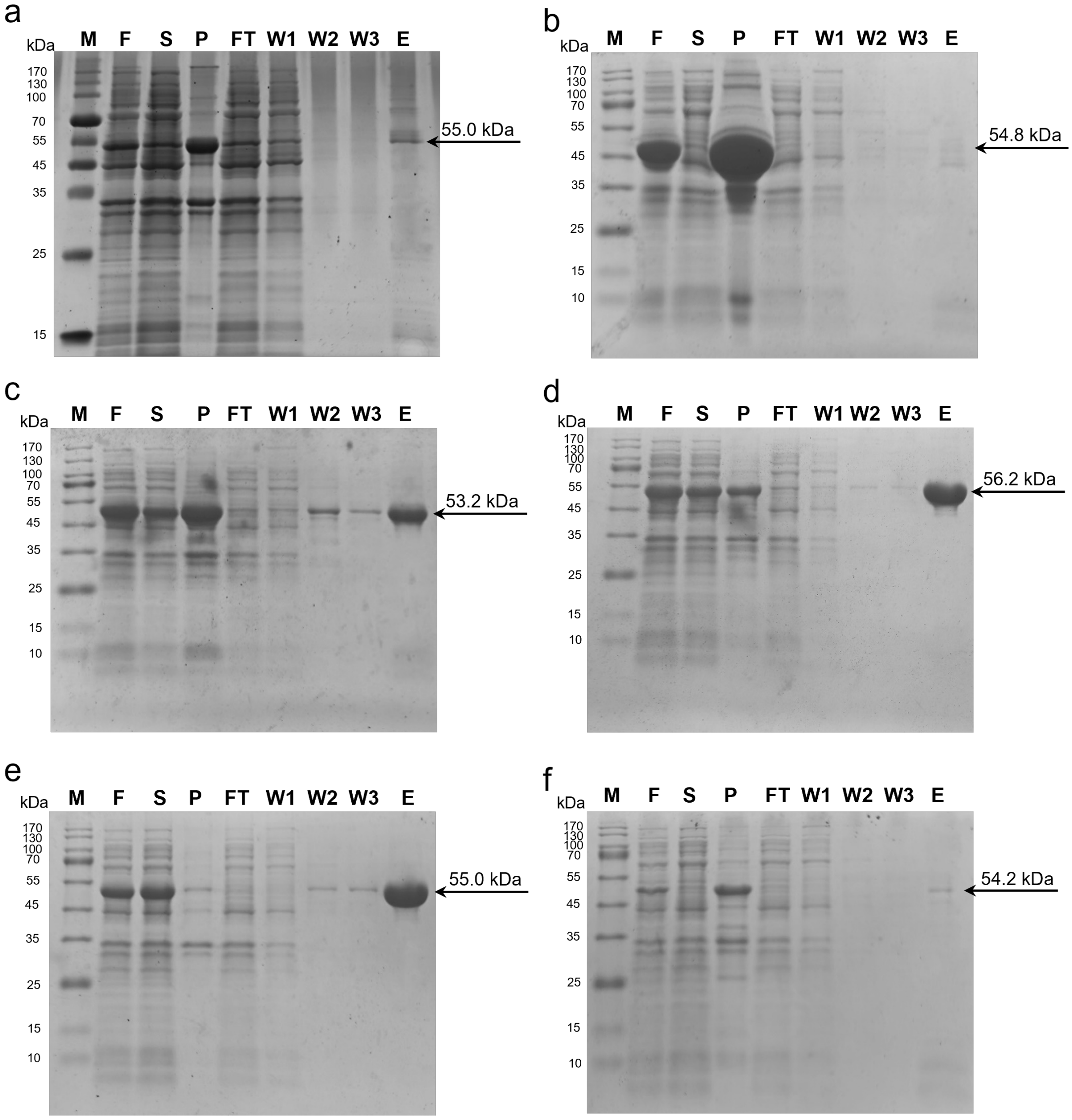

Supplementary Figure 16. SDS-PAGE analysis of CSO enzymes purified by affinity chromatography. a-f show the SDS-PAGE results of CSO2, PpCSO, MgpCSO, PgCSO, SsCSO, and TkCSO, respectively. Notes: F: fresh culture supernatant; S: supernatant after centrifugation; P: pellet after centrifugation; FT: flow-through from nickel column; W1: wash fraction 1 from nickel column; W2: wash fraction 2 from nickel column; W3: wash fraction 3 from nickel column; E: elution of protein using Elution buffer; M: protein molecular weight marker. The molecular weight indicated by the arrow on the right side of each panel was calculated using the molecular weight calculation tool at https://www.bioinformatics.org/sms/prot_mw.html.

Supplementary Table 1. Ability of the models to rank mutants on the k_{cat}, K_m, and k_{cat}/K_m datasets

SCC of CataPro/UniKP/DLKcat	N=20		N=30	
	**mean**	**medium**	**mean**	**Medium**
k_{cat}	0.284/-0.016/-0.061	0.265/-0.029/0.012	0.177/-0.076/0.027	0.161/-0.113/0.081
K_m	0.123/0.073/0.048	0.147/0.044/0.078	0.166/0.058/0.073	0.160/-0.040/0.053
k_{cat}/K_m	0.299/0.018/-0.099	0.317/0.010/-0.095	0.169/-0.018/-0.093	0.169/-0.011/-0.066

Note: The values in the table represent the SCC achieved by CataPro/UniKP/DLKcat.

Supplementary Table 2. Accuracy of the models in selecting the better-performing mutant from two mutants on the k_{cat}, K_m, and k_{cat}/K_m datasets

Accuracy of CataPro/UniKP/DLKcat	N=20		N=30	
	**mean**	**medium**	**mean**	**Medium**
k_{cat}	0.604/0.494/0.480	0.600/0.490/0.508	0.560/0.471/0.509	0.555/0.457/0.532
K_m	0.543/0.525/0.516	0.551/0.518/0.527	0.558/0.519/0.521	0.554/0.484/0.514
k_{cat}/K_m	0.604/0.505/0.462	0.613/0.497/0.458	0.542/0.493/0.463	0.546/0.496/0.474

Note: The values in the table represent the accuracy achieved by CataPro/UniKP/DLKcat.

Supplementary Table 3. Performance of the models in the global analysis of mutation effects.

Performance of CataPro/UniKP/DLKcat	N=20		N=30	
	**PCC**	**RMSE**	**PCC**	**RMSE**
k_{cat}	0.055/0.000/-0.002	1.081/1.204/1.264	0.034/-0.005/0.001	1.088/1.085/1.109
K_m	0.005/0.010/0.005	0.977/1.000/0.977	0.007/0.012/0.007	0.924/0.997/0.924
k_{cat}/K_m	0.037/0.001/-0.001	1.391/1.443/1.417	0.022/0.002/0.001	1.491/1.506/1.375

Note: The values in the table represent the PCC and RMSE achieved by CataPro/UniKP/DLKcat.

Supplementary Table 4. The SCC achieved by k_{cat}, K_m, and k_{cat}/K_m models of CataPro on the DMS datasets

		Fold-1	Fold-2	Fold-3	Fold-4	Fold-5	Fold-6	Fold-7	Fold-8	Fold-9	Fold-10	Ensemble
EcTL	k_{cat}	0.360	0.319	0.410	0.364	0.367	0.438	0.363	0.413	**0.392**	0.352	0.399
	K_m	0.172	0.338	0.344	0.313	0.323	**0.361**	0.093	0.007	0.380	0.277	0.296
	k_{cat} / K_m	0.390	0.396	0.440	0.309	0.335	0.374	0.203	**0.437**	0.373	0.384	0.398
TmIGPS	k_{cat}	0.467	**0.412**	0.471	0.469	0.447	0.469	0.477	0.405	0.395	0.439	0.472
	K_m	0.288	0.109	0.158	**0.145**	0.125	0.185	0.140	0.098	0.156	0.081	0.163
	k_{cat} / K_m	0.367	**0.426**	0.390	0.410	0.435	0.421	0.418	0.433	0.330	0.379	0.430
TtIGPS	k_{cat}	**0.474**	**0.419**	**0.449**	**0.492**	**0.450**	**0.456**	**0.518**	**0.404**	**0.458**	**0.515**	0.495
	K_m	**0.184**	**0.156**	**0.248**	**0.307**	**0.297**	**0.191**	**0.269**	**0.187**	**0.230**	**0.062**	0.235
	k_{cat} / K_m	**0.432**	**0.457**	**0.404**	**0.450**	**0.415**	**0.419**	**0.451**	**0.476**	**0.428**	**0.397**	0.465
SsIGPS	k_{cat}	0.478	0.514	**0.457**	0.512	0.479	0.440	0.556	0.479	0.532	0.505	0.535
	K_m	0.340	0.283	**0.257**	0.409	0.373	0.253	0.214	0.250	0.321	0.145	0.302
	k_{cat} / K_m	0.358	**0.447**	0.415	0.489	0.434	0.426	0.434	0.462	0.393	0.386	0.456

Note:

1. All SCC values for the K_m dataset are negative. To facilitate comparison, the SCC values for the K_m dataset are presented as absolute values.

2. **Bolded** values indicate SCC achieved by models in which no protein with sequence similarity greater than 0.5 to the test enzyme was included in the training set.

Supplementary Table 5. Relative activities of candidate enzymes discovered during enzyme mining and of SsCSO mutants obtained during enzyme modification

Step	CSOs	Relative Activity
Enzyme mining	CSO2	1.00
	PgCSO	0.20
	TkCSO	0.53
	MgpCSO	0.73
	PpCSO	1.13
	SsCSO	19.53
First round of enzyme modification for SsCSO	A43N	5.27
	H250W	6.84
	K134W	8.99
	V58W	27.93
	M351F	29.30
	T216M	30.28
Second round of enzyme modification for SsCSO	T216M-M351F-A305D	6.45
	T216M-M351F-S280F	32.62
	T216M-M351F-S301M	38.68
	T216M-M351F-V279M	52.15
	T216M-M351F-Q100G	61.71
	T216M-M351F-V384G	65.23

Note: The relative activity values in this table represent the activities of candidate enzymes or mutants relative to CSO2.

Supplementary Table 6. The hyperparameter options we tested for CataPro, with the final parameters marked in **bold**.

	Learning rate	Batch size	Dropout rate
Kcat	**0.00001**,0.0001,0.001,0.01	**8**,16,32,64,128	**0.0**,0.1,0.2,0.3,0.4
Km	**0.00001**,0.0001,0.001,0.01	8,**16**,32,64,128	0.0,**0.1**,0.2,0.3,0.4
Kcat/Km	0.00001,0.0001,0.001,**0.01**	8,**16**,32,64,128	0.0,**0.1**,0.2,0.3,0.4

Supplementary Table 7. The hyperparameter options we tested for UniKP, with the final parameters marked in **bold**.

	n_estimators	max_depth	min_samples_split	min_samples_leaf	max_features
Kcat	100,150,**200**,250	40,**80**,120,160,200	1,2,**4**,8	1,2,**4**,8	**1.0**,0.8,0.6,0.4
Km	100,150,200,**250**	40,80,120,160,**200**	1,**2**,4,8	1,**2**,4,8	**1.0**,0.8,0.6,0.4
Kcat/Km	100,150,**200**,250	**40**,80,120,160,200	1,2,**4**,8	**1**,2,4,8	1.0,**0.8**,0.6,0.4

Supplementary Table 8. The hyperparameter options we tested for DLKcat, with the final parameters marked in **bold**.

	radius	ngram	dim
Kcat	0,1,**2**	**1**,2,3	5,**10**,20
Km	0,1,**2**	**1**,2,3	5,**10**,20
Kcat/Km	0,1,**2**	**1**,2,3	5,**10**,20

Supplementary Table 9. The reagents used in the experiment and their sources

Name	Source
PrimeSTAR GXL DNA Polymerase	Takara
Hieff Clone® Plus One Step Cloning Kit	Yeasen
FastDigest DpnI	Thermo Scientific
DNA purification kit	Simgen
PAGE gel quick preparation kit	Epizyme Biotech
Tris	Macklin
2M HCl	Bolinda
peptone	Oxiod
yeast powder	Oxiod
agarose	Oxiod
IPTG	Aladdin
$FeCl_2 \cdot 4H_2O$	Macklin
Kanamycin sulfate	Aladdin
2,4-dinitrophenylhydrazine	Aladdin
NaOH	Sinopharm Chemical Reagent Co., Ltd
NaCl	Sinopharm Chemical Reagent Co., Ltd
Imidazole	Aladdin
Na_2HPO_4	Aladdin
NaH_2PO_4	Aladdin
Na_2CO_3	Aladdin
$NaHCO_3$	Aladdin
4-Vinyl Guaiacol	Aladdin

Supplementary Table 10. Primers for enzymes and mutants

Site	Primer Direction	Primer Sequence
S301M	Forward	CACCTCGATCTGTGCCTGATGGACACCAATGCTTTTGGTTT
S301M	Reverse	GCACAGATCGAGGTGAAC
A43N	Forward	GACGGCGCTTTCTTTCGCAACGTTCCAGATCCAGCTCATCC
A43N	Reverse	AAAGAAAGCGCCGTCAATC
A305D	Forward	TGCCTGTCTGACACCAATGATTTTGGTTTCATGCGCGAGGC
A305D	Reverse	GGTGTCAGACAGGCACAG
S280F	Forward	AAAGGTCGTAATGGTGTGTTTGCTTTCCATCTGGTTAACGC
S280F	Reverse	ACCATTACGACCTTTAAACC
V384G	Forward	CCACTGCCGGGTGGTCCGGGAGGTGTTGCGTTTAACGCTCT
V384G	Reverse	ACCACCCGGCAGTGGCGGAC
V279M	Forward	TTTAAAGGTCGTAATGGTATGTCTGCTTTCCATCTGGTTAAC
V279M	Reverse	ATTACGACCTTTAAACCAGC
K134W	Forward	GGTCGCCTCCTCATGACCTGGGAAGATGGCCTCGGCTACCAG
K134W	Reverse	CATGAGGAGGCGACCACC
H250W	Forward	GGCGGTGCTCACTGGGCTTGGCAACAGGACCTGGAGTCTTG
H250W	Reverse	CCAGTGAGCACCGCCAGC
Q100G	Forward	CGTCGTGCTCTGTTTGGCGGATACCGTAACCCGTTCACCGAC
Q100G	Reverse	AAACAGAGCACGACGCGCAT
V58W	Forward	ATGTTCGACGACGACATCTGGCTCTCTGGTGATGGCAT
V58W	Reverse	GATGTCGTCGTCGAACATCGGCGGATG
M351F	Forward	GTTGGCCCACCGGGTGATTTTCCACGCCTCCGTGACG
M351F	Reverse	ACCCGGTGGGCCAACCAG
T216M	Forward	GATCAGCCGTACTGCTCTATGATCCATGACTTCGCTATC
T216M	Reverse	GCAGTACGGCTGATCGAAC
