# Supplementary material for: Robust enzyme discovery and engineering with deep learning using CataPro
Source: Nat Commun. 2025 Mar 20;16:2736. doi: 10.1038/s41467-025-58038-4 (PMC11923063; doi:10.1038/s41467-025-58038-4)
Supplement: Supplementary file 2 — Description of Additional Supplementary Files [file 41467_2025_58038_MOESM2_ESM.pdf]

Description of Additional Supplementary Files

File Name: Supplementary Data 1
Description: Protein sequences of the tested CSO enzymes.

File Name: Supplementary Data 2
Description: Codon-optimized DNA sequences of the tested CSO enzymes generated using DNAworks v2 with the *E. coli* codon table.
