# Supplementary material for: Robust enzyme discovery and engineering with deep learning using CataPro
Source: Nat Commun. 2025 Mar 20;16:2736. doi: 10.1038/s41467-025-58038-4 (PMC11923063; doi:10.1038/s41467-025-58038-4)
Supplement: Supplementary file 5 — Reporting Summary [file 41467_2025_58038_MOESM5_ESM.pdf]

nature portfolio

Corresponding author(s): Liangzhen Zheng (zhenglz@zelixir.com)

Last updated by author(s): Feb 4, 2025

Reporting Summary

Nature Portfolio wishes to improve the reproducibility of the work that we publish. This form provides structure for consistency and transparency in reporting. For further information on Nature Portfolio policies, see our Editorial Policies and the Editorial Policy Checklist.

Statistics

For all statistical analyses, confirm that the following items are present in the figure legend, table legend, main text, or Methods section.

n/a	Confirmed	
☐	☒	The exact sample size (*n*) for each experimental group/condition, given as a discrete number and unit of measurement
☐	☒	A statement on whether measurements were taken from distinct samples or whether the same sample was measured repeatedly
☒	☐	The statistical test(s) used AND whether they are one- or two-sided<br>*Only common tests should be described solely by name; describe more complex techniques in the Methods section.*
☒	☐	A description of all covariates tested
☐	☒	A description of any assumptions or corrections, such as tests of normality and adjustment for multiple comparisons
☐	☒	A full description of the statistical parameters including central tendency (e.g. means) or other basic estimates (e.g. regression coefficient) AND variation (e.g. standard deviation) or associated estimates of uncertainty (e.g. confidence intervals)
☒	☐	For null hypothesis testing, the test statistic (e.g. *F*, *t*, *r*) with confidence intervals, effect sizes, degrees of freedom and *P* value noted<br>*Give P values as exact values whenever suitable.*
☒	☐	For Bayesian analysis, information on the choice of priors and Markov chain Monte Carlo settings
☒	☐	For hierarchical and complex designs, identification of the appropriate level for tests and full reporting of outcomes
☐	☒	Estimates of effect sizes (e.g. Cohen's *d*, Pearson's *r*), indicating how they were calculated

Our web collection on statistics for biologists contains articles on many of the points above.

Software and code

Policy information about availability of computer code

Data collection	Custom code used in this study is publicly available at https://github.com/zchwang/CataPro.
Data analysis	Custom code used in this study is publicly available at https://github.com/zchwang/CataPro.

For manuscripts utilizing custom algorithms or software that are central to the research but not yet described in published literature, software must be made available to editors and reviewers. We strongly encourage code deposition in a community repository (e.g. GitHub). See the Nature Portfolio guidelines for submitting code & software for further information.

Data

Policy information about availability of data

All manuscripts must include a data availability statement. This statement should provide the following information, where applicable:

- Accession codes, unique identifiers, or web links for publicly available datasets
- A description of any restrictions on data availability
- For clinical datasets or third party data, please ensure that the statement adheres to our policy

The information for the ten-fold cross-validation datasets that we created is sourced from BRENDA [https://www.brenda-enzymes.org/], SABIO-RK [http://sabio.h-its.org/], UniProt [https://www.uniprot.org/], and PubChem [https://pubchem.ncbi.nlm.nih.gov/]. The TAL homologue dataset and TAL engineering dataset are collected from https://doi.org/10.1038/s41467-023-44113-1 and https://doi.org/10.1093/bib/bbae387. The DERA dataset and BH1352 dataset are sourced from 10.1126/science.1063601 and 10.1074/jbc.RA119.011363, respectively. The mutation dataset of EcTL is from https://doi.org/10.1093/molbev/msu081, and the mutation datasets of TmIGPS, TtIGPS, and SsIGPS are all from https://doi.org/10.1038/ncomms14614. The unbiased ten-fold cross-validation datasets we created

are publicly available in the Zenodo repository at https://zenodo.org/records/14894710 and the GitHub repository at https://github.com/zchwang/CataPro. Source data are provided with this paper. Unless otherwise stated, all data supporting the results of this study can be found in the article, supplementary, and source data files.

Research involving human participants, their data, or biological material

Policy information about studies with human participants or human data. See also policy information about sex, gender (identity/presentation), and sexual orientation and race, ethnicity and racism.

Reporting on sex and gender	N/A
Reporting on race, ethnicity, or other socially relevant groupings	N/A
Population characteristics	N/A
Recruitment	N/A
Ethics oversight	N/A

Note that full information on the approval of the study protocol must also be provided in the manuscript.

Field-specific reporting

Please select the one below that is the best fit for your research. If you are not sure, read the appropriate sections before making your selection.

[x] Life sciences [ ] Behavioural & social sciences [ ] Ecological, evolutionary & environmental sciences

For a reference copy of the document with all sections, see nature.com/documents/nr-reporting-summary-flat.pdf

Life sciences study design

All studies must disclose on these points even when the disclosure is negative.

Sample size	All entries collected from the public databases were cleaned to generate the final datasets. In total, there are 27,658 samples with kcat values, 42,018 samples with Km values, and 25,831 samples with both kcat and Km values. For each of kcat, Km, and kcat/Km datasets, we clustered samples based on a 0.4 protein sequence similarity and divided them into ten groups, creating an unbiased ten-fold cross-validation dataset. We trained the models on the unbiased ten-fold cross-validation datasets for kcat, Km, and kcat/Km.
Data exclusions	During data collection, entries with enzymes having multiple UniProt IDs were removed. Additionally, entries with incomplete information were excluded.
Replication	The code is clear, concise, and user-friendly, allowing users to follow the prompts to run the program and reproduce the results.
Randomization	We tested the models in various scenarios, including datasets with random splits and unbiased ten-fold cross-validation. We found that random splitting can lead to significant bias, resulting in overly optimistic model evaluations. Therefore, in our paper, we emphasize the importance of training and evaluating models on unbiased datasets.
Blinding	In the ten-fold unbiased cross-validation datasets we constructed, the protein sequence similarity between each component was less than 0.4. Therefore, training and evaluating the models on the unbiased datasets actually simulates a real-world application scenario, where the enzymes in the real world may differ significantly from those in the training set. Additionally, we introduced several external test sets to evaluate the generalization of models, and the sequence identities between the enzymes in these test sets and those in the training set are mentioned in the main text.

Reporting for specific materials, systems and methods

We require information from authors about some types of materials, experimental systems and methods used in many studies. Here, indicate whether each material, system or method listed is relevant to your study. If you are not sure if a list item applies to your research, read the appropriate section before selecting a response.

Materials & experimental systems

n/a	Involved in the study
☒	☐ Antibodies
☒	☐ Eukaryotic cell lines
☒	☐ Palaeontology and archaeology
☒	☐ Animals and other organisms
☒	☐ Clinical data
☒	☐ Dual use research of concern
☒	☐ Plants

Methods

n/a	Involved in the study
☒	☐ ChIP-seq
☒	☐ Flow cytometry
☒	☐ MRI-based neuroimaging

Plants

Seed stocks	N/A
Novel plant genotypes	N/A
Authentication	N/A
