# Supplementary figures and images for: Robust enzyme discovery and engineering with deep learning using CataPro
Source: Nat Commun. 2025 Mar 20;16:2736. doi: 10.1038/s41467-025-58038-4 (PMC11923063; doi:10.1038/s41467-025-58038-4)

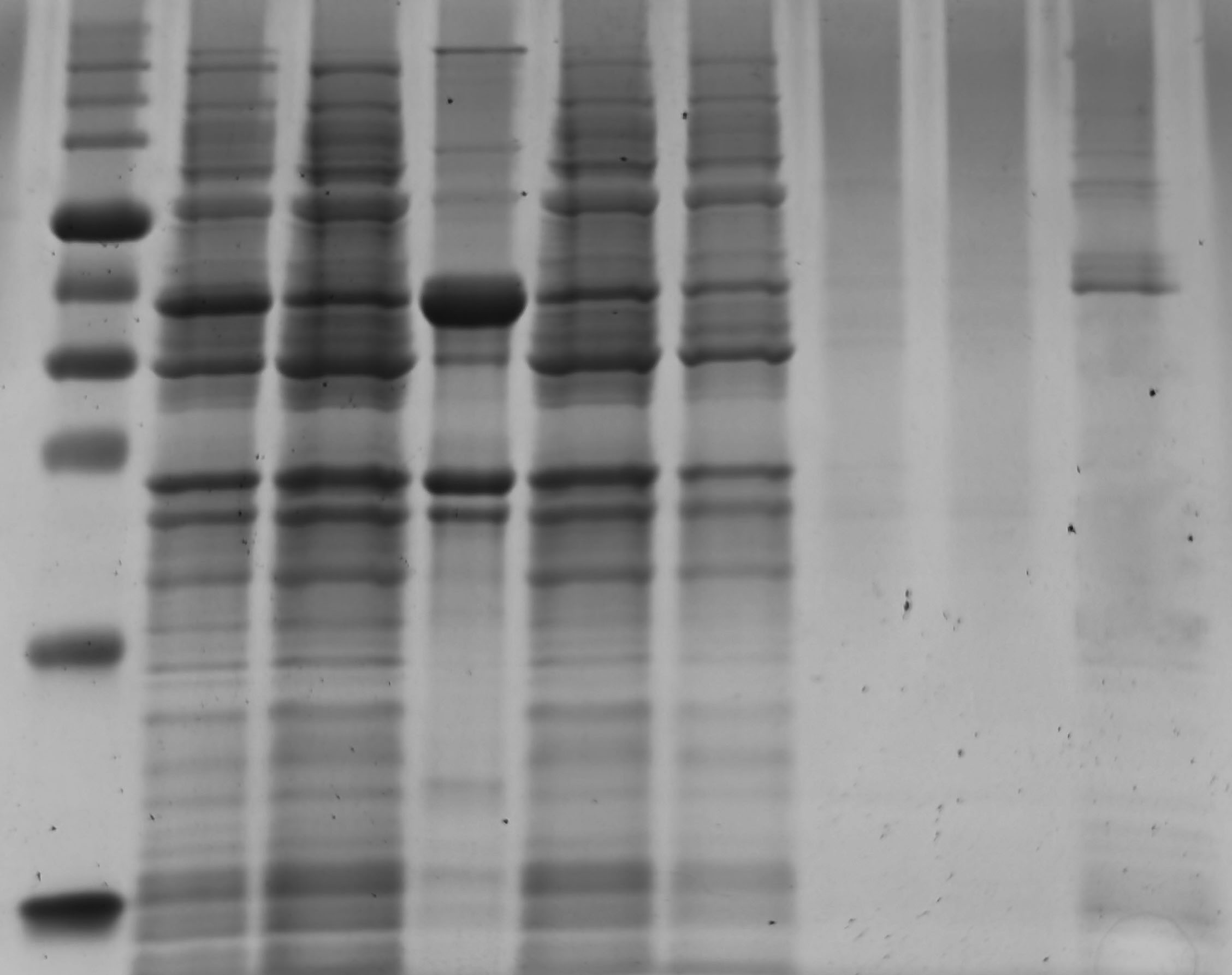

Supplement: Supplementary file 7 — Source Data [file 41467_2025_58038_MOESM7_ESM.zip › Source Data/SDS-PAGE_images/CSO2.jpg]

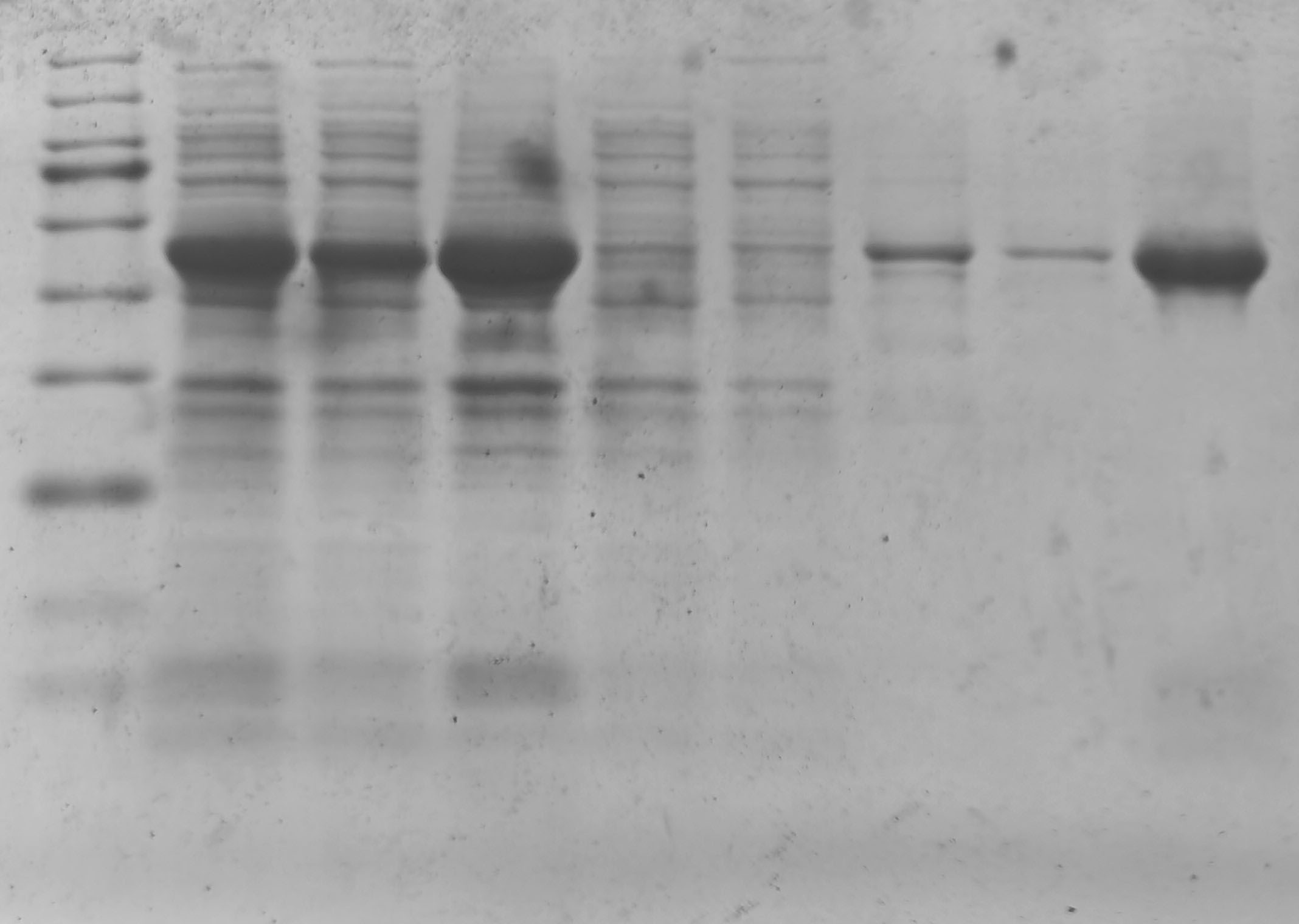

Supplement: Supplementary file 7 — Source Data [file 41467_2025_58038_MOESM7_ESM.zip › Source Data/SDS-PAGE_images/MgpCSO.jpg]

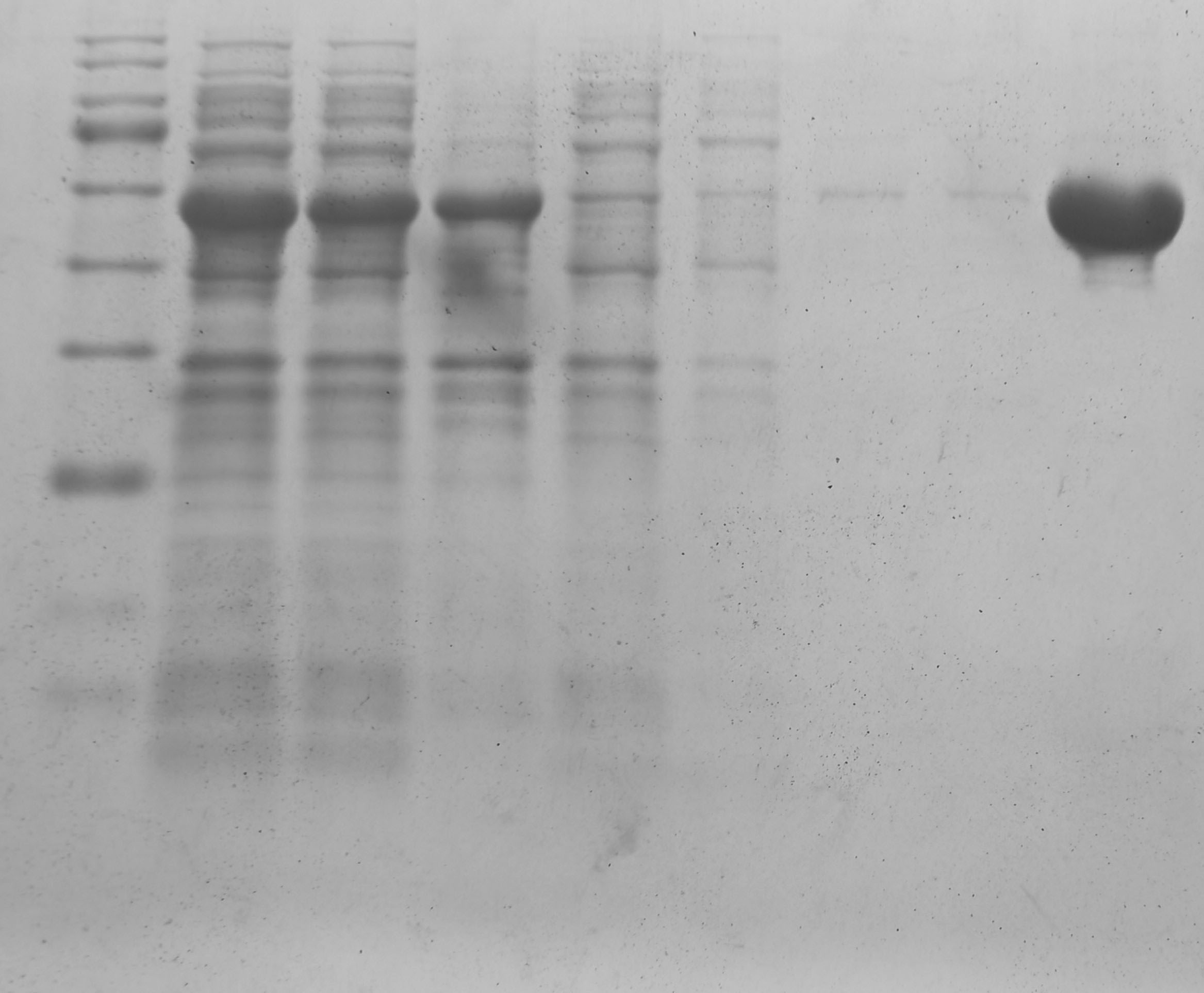

Supplement: Supplementary file 7 — Source Data [file 41467_2025_58038_MOESM7_ESM.zip › Source Data/SDS-PAGE_images/PgCSO.jpg]

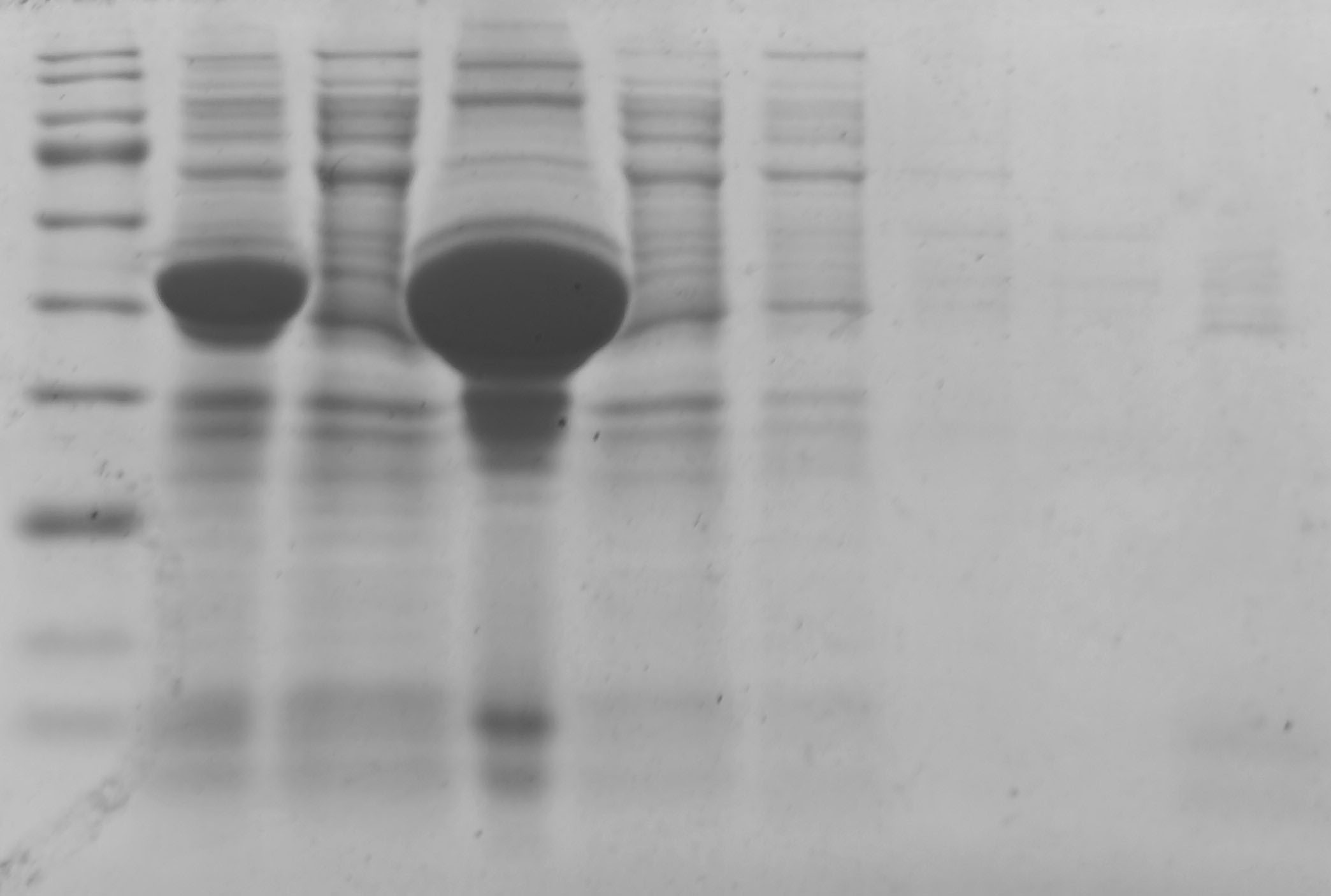

Supplement: Supplementary file 7 — Source Data [file 41467_2025_58038_MOESM7_ESM.zip › Source Data/SDS-PAGE_images/PpCSO.jpg]

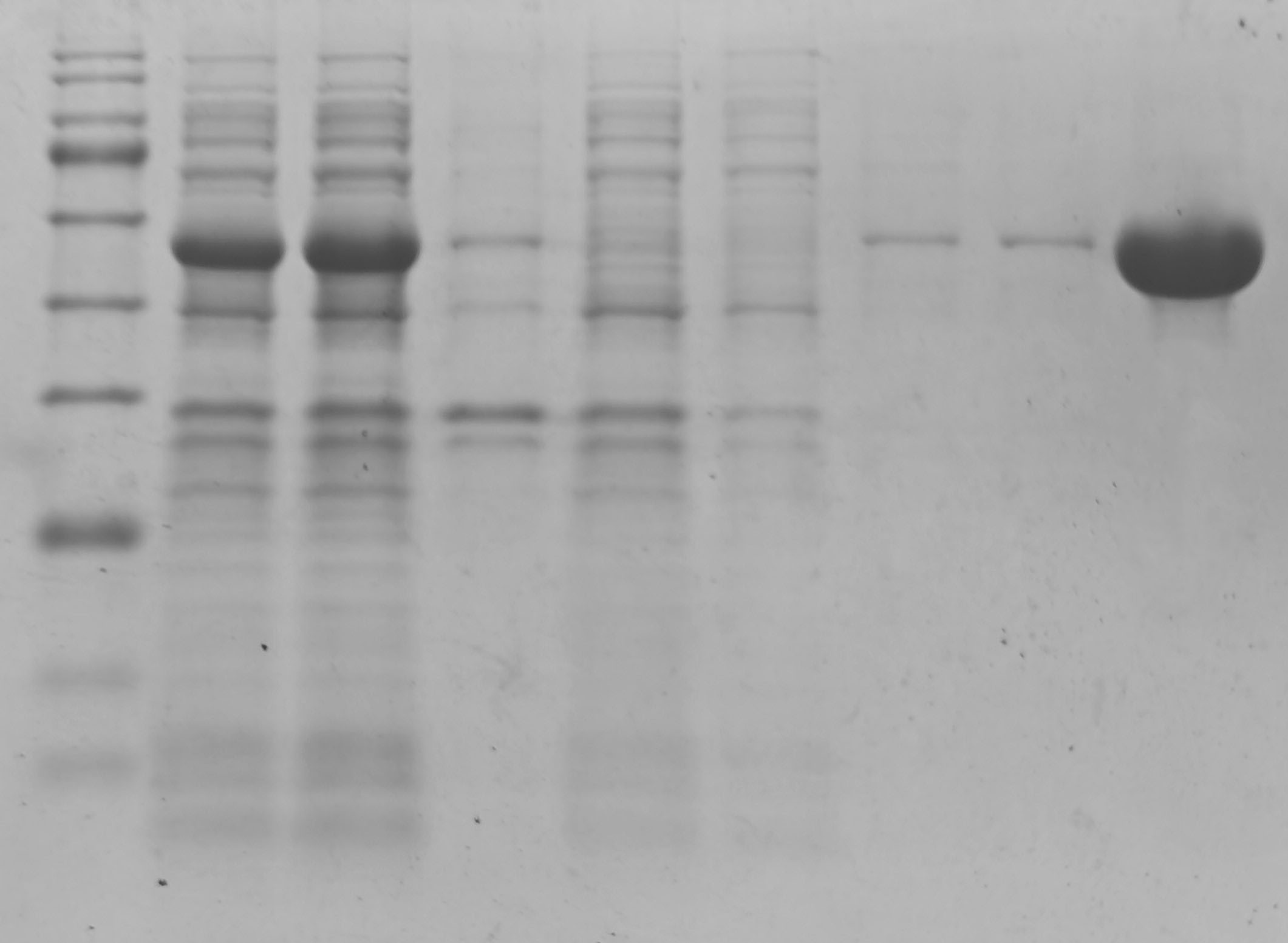

Supplement: Supplementary file 7 — Source Data [file 41467_2025_58038_MOESM7_ESM.zip › Source Data/SDS-PAGE_images/SsCSO.jpg]

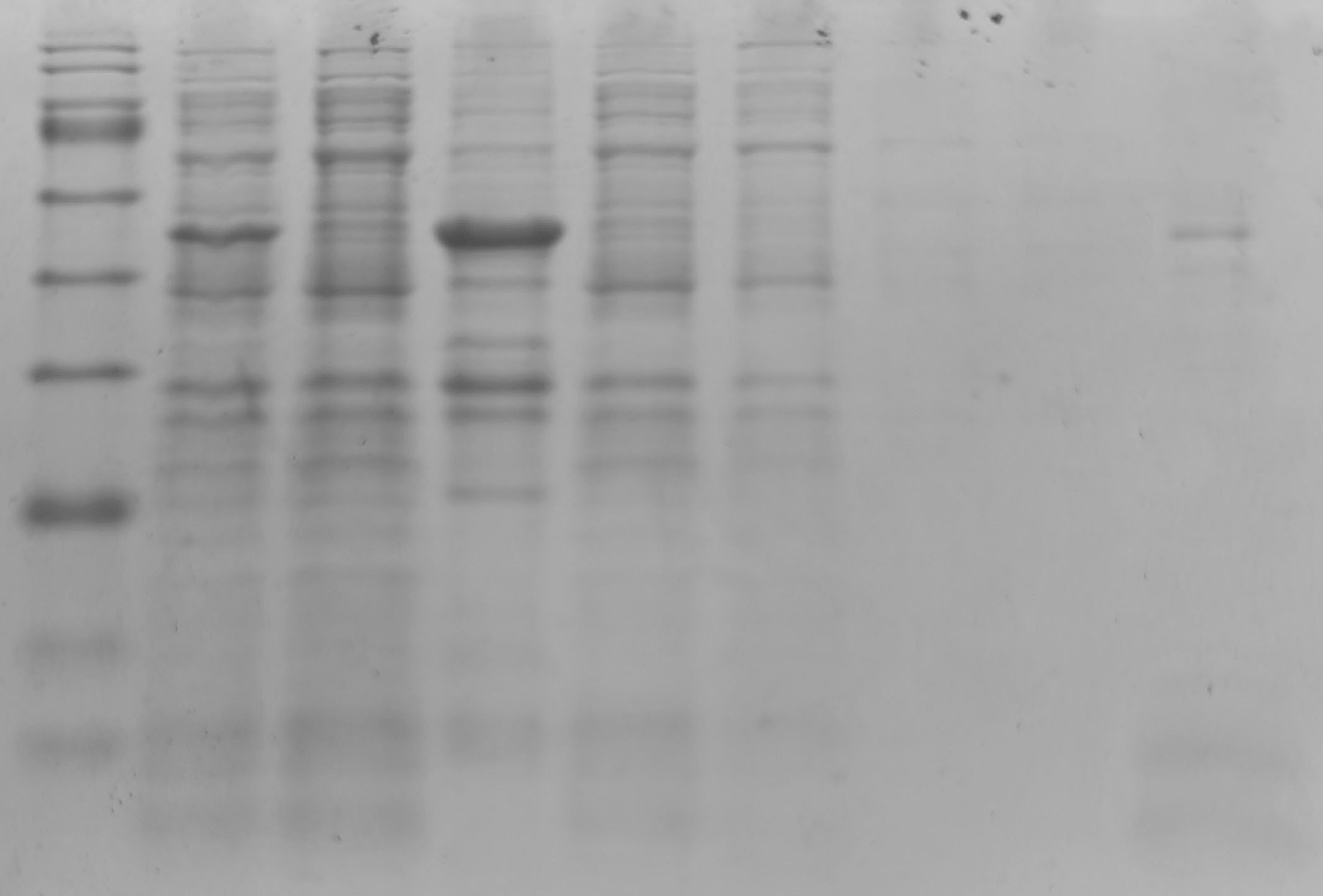

Supplement: Supplementary file 7 — Source Data [file 41467_2025_58038_MOESM7_ESM.zip › Source Data/SDS-PAGE_images/TkCSO.jpg]
